# Microbial Tyrosinases: Promising Enzymes for Pharmaceutical, Food Bioprocessing, and Environmental Industry

**DOI:** 10.1155/2014/854687

**Published:** 2014-05-06

**Authors:** Kamal Uddin Zaidi, Ayesha S. Ali, Sharique A. Ali, Ishrat Naaz

**Affiliations:** ^1^Molecular Biotechnology Laboratory, Centre for Scientific Research & Development, People's University, Bhanpur, Bhopal 462010, India; ^2^Department of Biotechnology, Saifia College of Science, Bhopal 462001, India

## Abstract

Tyrosinase is a natural enzyme and is often purified to only a low degree and it is involved in a variety of functions which mainly catalyse the *o*-hydroxylation of monophenols into their corresponding *o*-diphenols and the oxidation of *o*-diphenols to *o*-quinones using molecular oxygen, which then polymerizes to form brown or black pigments. The synthesis of *o*-diphenols is a potentially valuable catalytic ability and thus tyrosinase has attracted a lot of attention with respect to industrial applications. In environmental technology it is used for the detoxification of phenol-containing wastewaters and contaminated soils, as biosensors for phenol monitoring, and for the production of L-DOPA in pharmaceutical industries, and is also used in cosmetic and food industries as important catalytic enzyme. Melanin pigment synthesized by tyrosinase has found applications for protection against radiation cation exchangers, drug carriers, antioxidants, antiviral agents, or immunogen. The recombinant *V. spinosum* tryosinase protein can be used to produce tailor-made melanin and other polyphenolic materials using various phenols and catechols as starting materials. This review compiles the recent data on biochemical and molecular properties of microbial tyrosinases, underlining their importance in the industrial use of these enzymes. After that, their most promising applications in pharmaceutical, food processing, and environmental fields are presented.

## 1. Introduction

Tyrosinase (EC 1.14.18.1) is a copper-containing monooxygenase catalyzing the* o*-hydroxylation of monophenols to the corresponding catechols (monophenolase or cresolase activity) and the oxidation of monophenols to the corresponding* o*-quinones (diphenolase or catecholase activity). It is involved in the biosynthesis of melanin and catalyses the* ortho*-hydroxylation of tyrosine (monophenol) to 3,4-dihydroxyphenylalanine or DOPA (*o*-diphenol) and the oxidation of DOPA to dopaquinone (*o*-quinone). This* o*-quinone can then be transformed into melanin pigments through a series of enzymatic and nonenzymatic reaction [1, 2]. Not only the physiological substrates tyrosine and L-DOPA but also various other phenols and diphenols are converted by tyrosinase to the corresponding diphenols and quinones, respectively. Thus, in general, tyrosinase catalyses both the* ortho*-hydroxylation of monophenols (cresolase or monophenolase activity) and the two-electron oxidation of* o*-diphenols to* o*-quinones (catecholase or diphenolase activity). These reactions take place under concomitant reduction of dioxygen to water. The mechanism by which an oxygen atom is transferred to the phenol substrate is proposed to begin with either a *μ* − *η* ^2^ : *η* ^2^-peroxodicopper (II) intermediate or a bis-*μ*-oxodicopper (III) intermediate. These intermediates have characteristic resonance Raman spectra. Synthetic studies provide models of both complexes and evidence for rapid equilibrium between the two forms [3]. The monophenol hydroxylase and diphenoloxidase activities of tyrosinases are the basis for many industrial biotechnological applications like in environmental technology for the detoxification of phenol-containing waste waters and contaminated soils as a construction of a biosensor for the detaction of phenolic compound [4] and in pharmaceutical industries for the production of* o*-diphenols (e.g., L-DOPA, dopamine for the treatment of Parkinson's disease) and also have been tested as a marker in melanoma patients [5] and as a target for the activation of prodrugs [6] in food industries for modification of food proteins via crosslinking affects [7]. Synthetic melanin is also used for protection against radiation (UV, X-ray, and gamma ray), cation exchangers, drug carriers, antioxidants, antiviral agents, or immunogen. There is considerable information representing the great potential of this enzyme for food, medicine, and agricultural industries as well as for analytical and environmental purposes [8–10].

The best-characterized tyrosinases are derived from* Streptomyces glaucescens* and the fungi* Neurospora crassa* and* Agaricus bisporus.* In fungi and vertebrates, tyrosinase catalyzes the initial step in the formation of the pigment melanin form tyrosine. In plants, the physiological substrates are variety of phenolics and tyrosinase oxidizes them in the browning pathway observed when tissues are injured. The enzyme extracted from the champignon mushroom* Agaricus bisporus *is highly homologous with the mammalian ones, and this renders it well suited as a model for studies on melanogenesis. In fact, almost all studies on tyrosinase inhibition conducted so far have used mushroom tyrosinase because the enzyme is commercially available [11]. It was investigated by Molloy et al. [12] recently that bacterial tyrosinase from* Ralstonia solanacearum* can be modified through engineering which in turn is used for the improved catalytic efficiency towards D-tyrosine using random and site directed mutagenesis. Mushrooms are considered a cheap source of the tyrosinase. Mushroom tyrosinase from* Agaricus bisporus *is a tetramer of about 120 KDa with monomeric isoforms with molecular masses of 30 KDa. Furthermore, microbial tyrosinase (mushroom tyrosinase) like mammalian tyrosinase has a tetrameric structure and can be used for clinical purposes [13].

All these features have made microbial tyrosinases a suitable tool for today's pharmaceutical, food bioprocessing, and environmental technology. For different purposes, we review the potential applications of microbial tyrosinases and evaluate the state of knowledge about their isolation production, purification, sources, biochemical properties, and applications. We conclude that much more research is required in these areas as microbial tyrosinases can fulfill their industrial potential. Whereas tyrosinases are widely distributed in mushroom, plants, invertebrates, and mammals, much of the modern significance in the development of pharmaceutical, food bioprocessing, and environmental applications has focused on the use of microbial tyrosinases. This paper presents recent advances in tyrosinases from microbial origin with emphasis on their biochemical properties and discusses their current and potential applications.

## 2. Sources of Tyrosinase

Tyrosinase activities are widely distributed in all domains of life from microorganisms to mammals. Tyrosinases have been purified and their properties and functions have been extensively studied. They are found in whole cells, tissues from mushrooms, fruits, and vegetables and are mainly involved in the biosynthesis of melanins and other polyphenolic compounds.

### 2.1. Fungus as a Source of Tyrosinase

Different fungi have been investigated for the isolation of tyrosinase which has been obtained from* Agaricus bisporus* [14],* Neurospora crassa* [15, 16],* Amanita muscaria* [17],* Lentinula edodes* [18],* Aspergillus oryzae *[19], Portabella mushrooms,* Pycnoporus sanguineus* [20], and* Lentinula boryana* [21].

### 2.2. Bacteria as a Source of Tyrosinase

Streptomyces tyrosinases are the most thoroughly characterized enzymes of bacterial origin [22, 23]. Bacterial tyrosinase is involved in the melanin production and is normally extracellular in origin. The enzyme has been reported in other species such as* Rhizobium, Symbiobacterium thermophilum, Pseudomonas maltophilia, Sinorhizobium meliloti, Marinomonas mediterranea, Thermomicrobium roseum, Bacillus thuringiensis*,* Pseudomonas putida *[24–26],* Streptomyces castaneoglobisporus, Ralstonia solanacearum*, and* Verrucomicrobium spinosum* [27].

### 2.3. Plants as a Source of Tyrosinase

Tyrosinase from various fruits and vegetables has been studied and the enzyme has been extracted from Monastrell grape (Janovitz-Klapp et al. 1989), apple [28], sunflower seed [29], and* Solanum melongena *[30]. In plants, tyrosinase is localized in the chloroplasts of healthy plant tissues, whereas its substrates are contained in the vacuole.* Portulaca grandiflora* (Portulacaceae) is a potent source of tyrosinase [31–33]. It generally causes undesired enzymatic browning of farm products which subsequently leads to a significant decrease in the nutritional and market values.

## 3. Structural Properties of Tyrosinase

**Figure 1:**
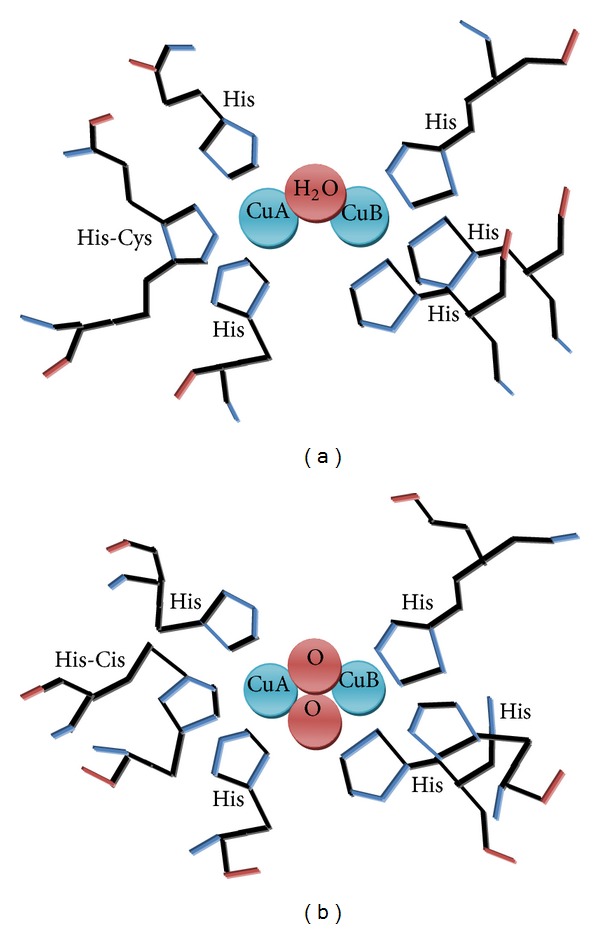
Structure of tyrosinase.

Tyrosinase is diverse in terms of its structural properties, tissue distribution, and cellular location as no common tyrosinase protein structure occurs across all species [34, 35]. The enzyme frequently differs to its primary structure, size, glycosylation pattern, and activation characteristics. However, all tyrosinases have a common binuclear type III copper centre (T3Cu), of two copper atoms, each coordinated by three histidine residues, within their active site. The copper pair present in their active site binds to atmospheric oxygen so as to catalyze two different kinds of enzymatic reactions (I)* ortho*-hydroxylation of monophenols and (II) oxidation of* o*-diphenols to* o*-quinones, similar to the coordination mode found in hemocyanins. In the crystal structures of these enzymes, six histidine residues, which are provided by a four-helix bundle in the copper-binding domain to the two copper ions (three histidine imidazoles for each copper ion, CuA and CuB), in the active site (Figures 1(a) and 1(b)). Tyrosinases and catechol oxidases are collectively termed polyphenol oxidases due to their similar activity with catechol oxidase. Mushroom tyrosinase is a tetrameric glycoprotein copper containing metalloenzyme with a molecular weight of 128 ± 6.4 kD and a subunit molecular weight of 32 kD [36]. Four tyrosinase genes of the common button mushroom,* Agaricus bisporus*, have been investigated to date. Wichers and coworkers have found two genes encoding two 64 kDa tyrosinases; ppo1 (Genebank accession number X85113) and ppo2 (AJ223816) were studied by Wichers et al, whereas the other two genes, which encode a 66 and 68 kDa tyrosinase ppo3 (GQ354801) and ppo4 (GQ354802), were recently reported by Wu et al. [37]. Unlike the fungal tyrosinase, human tyrosinase is a membrane-bound glycoprotein [38]. Experimental data suggests tyrosinase to be a monomeric protein with more than one isoform [13, 39, 40]. Similarly, bacterial tyrosinases from* Streptomyces* species are nonmodified monomeric proteins with a relatively low molecular mass of 30 kDa. These enzymes are secreted to the surrounding medium and are involved in extracellular melanin production. Tyrosinase exists in three forms in the catalytic cycle (met, oxy, and deoxy) with different binuclear copper structure of the active site which are involved in the biosynthesis of melanin [15, 16, 41, 42]. Microbial tyrosinases have been divided into five types according to the organization of domain and the possible requirement of a caddie protein for enzyme activity [43]. The necessity of a secondary helper protein (caddie protein) for secretion, correct folding, assembly of the copper atoms, and activity of the enzyme is common to tyrosinases of type I, for example, the enzyme from* S. casstaneoglobisporus* and* S. antibioticus*. Type II tyrosinases are small monomeric enzymes containing only the catalytic domain, which do not require additional helper proteins and are possibly secreted. An example is the tyrosinase from* B. Megaterium *[44]. Type III tyrosinases are represented by the enzyme from* Verrucomicrobium spinosum*. Like the fungal tyrosinases it carries a C-terminal domain whose removal led to about 100-fold higher activity [45]. This supports the theory that the role of the C-terminal extension in plant and fungal tyrosinases is to keep the enzyme in an inactive form inside the cell [46, 47]. Among the smallest bacterial tyrosinases reported (type IV) are the ones produced by* Streptomyces ingrifaciens* (18 kDa) and* Bacillus thuringiensis* (14 kDa) [24].

## 4. Biochemical Features of Microbial Tyrosinase

In this section we provide a brief outline of the biochemical characteristics of the most studied microbial tyrosinase; several scientists all over the world are working on the biochemical properties of the enzyme tyrosinase as depicted in Table 1. Tyrosinase has broad substrate specificity where the enzyme has a higher affinity for the L-isomers of the substrates than for the corresponding D-isomers [48]. Tyrosinase derived from* Streptomyces glaucescens, Neurospora crassa*, and* Agaricus bisporus *is best characterized. Tyrosinase catalyzes the formation of the pigment melanin from tyrosine whereas, in plants, phenolics act as a physiological substrate. Tyrosinases exist in immature, mature latent, and active isoforms [49, 50]. Kinetic studies of the steady state of the pathway show the lower catalytic efficiency of tyrosinase on monophenols than on* o*-diphenols [51–53]. Kinetic studies also show the activation of tyrosinase and the decrease in lag time due to the presence of reducing agents (cofactors, especially* o*-diphenol derivatives such as L-DOPA and (+)-catechin). Mammalian tyrosinase is found specially in melanocytes, which are highly specialized cells located in the skin, hair bulbs, and eyes which produce pigments. Mammalian tyrosinase goes through an extensive process of posttranslational modification which occurs in Golgi complex [54].

## 5. Isolation of Tyrosinases

Tyrosinases have been isolated from plants, fungi, actinomycetes, and bacteria. A homology search of the genome database of the filamentous fungus* Trichoderma reesei* identified a new* T. reesei* tyrosinase gene tyr2, encoding a protein with a putative signal sequence. The gene was over expressed in the native host under the strong cbh1 promoter, and the tyrosinase enzyme was secreted into the culture supernatant [63]. Isolation of tyrosinase requires ample of process, as it could not be isolated in sufficient quantities and purities for detailed structural studies and thus isolation of tyrosinase from bacteria is simple. For example, the* Streptomyces* tyrosinases are nonmodified monomeric protein with a low molecular mass of 30 kDa and are secreted to the surrounding medium, where they are involved in extracellular melanin production. However, the relative activities, optimum pH of activity, and molecular weight of these enzymes show considerable variations. Microbial tyrosinase is either produced by submerged culture or by solid-state fermentation. Quantitative assays are a powerful tool used for screening fungi for enzyme production as they are helpful in screening large number of fungal isolates and thus provide an insight into the conditions necessary to stimulate production in submerged culture. Halaouli et al. [20] studied the production of intracellular tyrosinases from various strains of* Pycnoporus cinnabarinus* and* Pycnoporus sanguineus*. Functionally expressed a tyrosinase gene from Streptomyces antibioticus in Escherichia coli under the control of an inducible bacteriophage T7 promoter. These cells produced melanin pigments on agar plates and in liquid culture when supplemented with copper and tyrosine [23].

## 6. Purification of Tyrosinase

**Figure 2:**
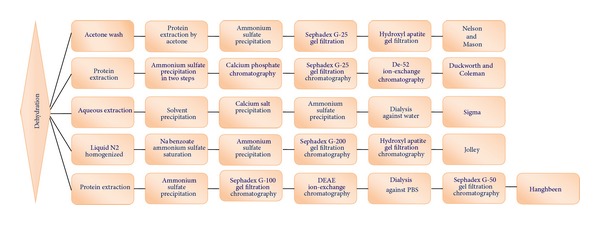
Purification scheme of tyrosinase.

Tyrosinases are natural enzymes and are often purified to only a low degree. Different methods have been used for the purification of microbial tyrosinases as depicted in Figure 2. The culture filtrate dehydrated with acetone or ammonium sulfate and calcium salt is added to precipitate the enzyme and other proteins [17]. The concentration of ammonium sulfate also plays an important role in the precipitation of the enzyme as studies have shown variation in concentration of ammonium sulphate varying from 35% to 70% saturated solution subsequently at two steps [64], 25–70% [30]. Although there are numerous methods for purifying tyrosinases from different sources, few methods are cited for its production especially from various species of mushroom [58, 65, 66]. For the purification of microbial tyrosinases various columns containing hydroxylapatite [67], DEAE-cellulose [68], or size exclusion gel [69] have been performed.

## 7. Medicinal Application of Tyrosinases

Tyrosinases are omnipresent in nature and are considered one of the fundamental enzymes involved in several biological functions and defense mechanism (especially in melanogenesis). Tyrosine-related melanogenesis is responsible for pigmentation of hair, skin, and eye in mammals, as pigmentation is a pivotal part of skin protection by UV radiation [70]. Abnormal melanin can result from the changes anywhere in the pathway of synthesis either due to abnormal tyrosinase or due to the deficits caused by the transfer of melanosomes to keratinocytes [71, 72]. The role of tyrosinase is different in different kingdoms, as in invertebrates they play a crucial task in defense and sclerotization reactions [73]. Whereas in microbial world its use is still unknown, melanin helps in the formation of reproductive organs and spores and in cell wall protection after physical damage [25].* Agaricus bisporus *[74],* Pycnoporus sanguineus* [20],* Aspergillus oryzae* [75],* Aspergillus flavipes* [76],* Neurospora crassa *[77], and* Lentinula edodes* [18] show tyrosinase activity and thus act as sources for its retrieval.

## 8. Pharmaceutical Importance of Tyrosinase in Melanin Biosynthesis

**Figure 3:**
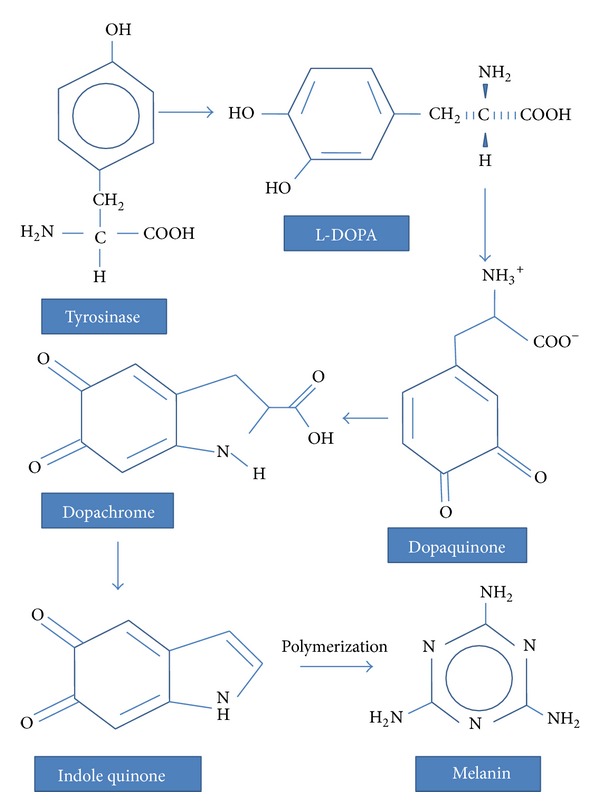
Melanin biosynthetic pathway.

Tyrosinase is known to be the key enzyme in melanin biosynthesis. Melanin is the most important pigment synthesized through a physiological process called melanogenesis in membrane-bound subcellular organelles, the melanosomes which remain present in black; dendritic cells of the skin are called melanocytes and their activity is the major determinant of the color of the hair and skin. Melanocytes are produced in the neural crest and travel to the basal layer of the epidermis and the hair matrices during embryogenesis. They play a pivotal role in protecting the skin from dreadful UV light by absorbing UV rays of sunlight and removing reactive oxygen species [78, 79]. Due to exposure to sunlight, the number of melanosomes increases, thus increasing their melanin content and their transfer to keratinocytes. Melanin is a class of compounds which is also found in the plant, animal, and Protista kingdoms, where it serves predominantly as a pigment and is synthesized within melanosomes, a membrane-bound granule [78]. The melanin biosynthetic pathway is outlined in Figures 3(a) and 3(b) where tyrosinase catalyses hydroxylation of the amino acid tyrosine to 3,4-dhydroxyphenylalanine (DOPA) by monophenolase action and oxidation of DOPA into* o*-dopaquinone by diphenolase action. This* o*-quinone is transformed into melanin in a series of nonenzymatic reactions [80, 81].

**Figure 4:**
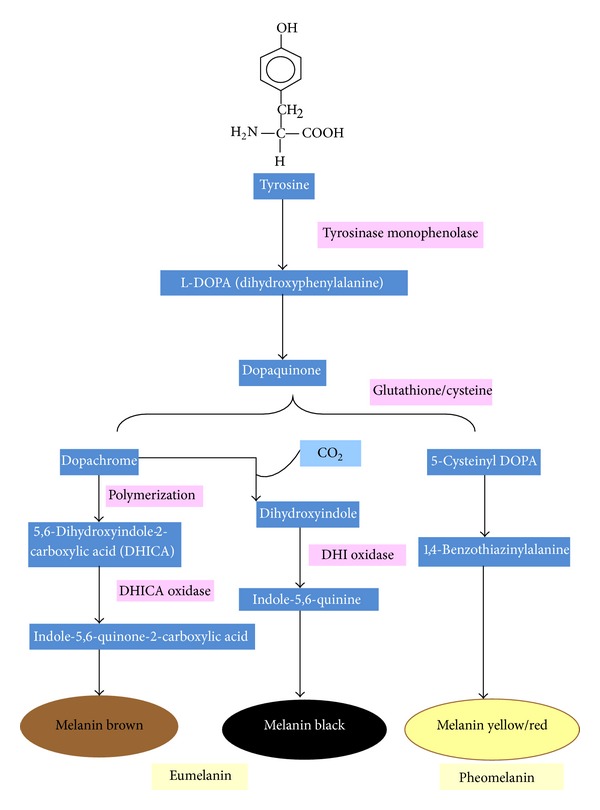
Production of different pigments by melanosomes.

There are two types of melanin pigments that can be produced by the melanocytes, namely, “eumelanin” (black or brown) and “pheomelanin” (red or yellow) as shown in Figure 4. Both amount and type genes operate under incomplete dominance [79].

**Figure 5:**
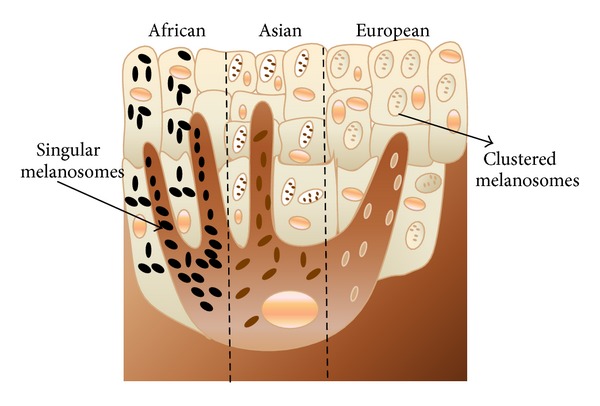
Structure or melanosome distribution for different racial groups.

Several genes expression that is MCIR, SLC45A2, ASIP, TYR, OCA2, and SLC24A5 is involved in the variation of skin tones in humans due to difference in fraction in melanin units between Europeans and Africans. Number of melanocytes cells are very much similar in individuals of different racial groups as shown in Figure 5. Thus the type of melanin production depends on the functioning of the melanocytes, people with darker skin are just genetically programmed to continuously produce higher levels of melanin even without exposure to UV light and their melanosomes remains singular, however in case of individuals with fair skin colour, melanosomes packed itself in the form of membrane-bound organelles [78–80].

Tyrosinases play an important role in melanogenesis (i.e., biosynthesis of melanin pigments also known as pigmentation). In the reaction, tyrosine is first oxidized to dopaquinone, which either cyclises to give a dihydroxyindole precursor of black or brownish eumelanins or reacts with cysteine to give a precursor of reddish brown pheomelanin [81]. Overactivity of this enzyme leads to overproduction of melanin leading to hyperpigmentation of the skin and underactivity leads to disorders such as vitiligo (depigmentation spots that occur of the skin) and whitening of hair. Some commercially available chemical and fungal derived skin-lightening agents have been proven to have chronic, cytotoxic, and mutagenic effects in humans [82, 83].

## 9. Microbial Tyrosinase for Manufacture of L-DOPA in Pharmaceutical Industries

**Figure 6:**
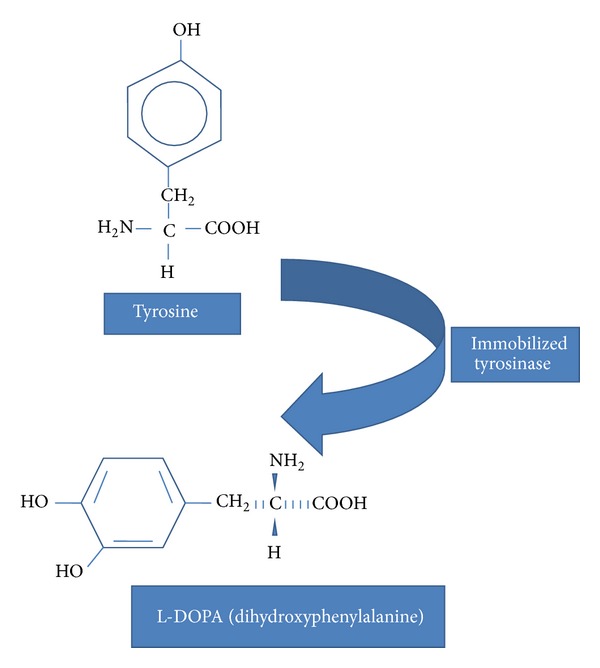
Industrial production of L-DOPA using of microbial tyrosinase.

L-Dihydroxy phenylalanine is a naturally occurring dietary supplement and psychoactive drug found in certain kinds of food and herbs (e.g.,* Mucuna pruriens* or velvet bean) and is synthesized from the amino acid L-tyrosine in the mammalian body and brain. L-DOPA is a precursor of the production of dopamine by the central nervous system. Thus, L-DOPA is used as a potent drug for the treatment of Parkinson's disease and is also used to control the myocardium neurogenic injury [84]. Production of L-DOPA using L-tyrosine as substrate and L-ascorbate as reducing agent with the enzyme tyrosinase (EC 1.14.18.1) as biocatalyst has been studied [85]. Manufacturing L-DOPA using microbial tyrosinases in batch reactors ranges from 1.44 to 54 mg [86]. But the production seems to be much less due to two factors; that is, firstly, addition of L-tyrosine is incomplete, with less than 30% of it being consumed during the process. Secondly, due to side reactions and reversible process intermediates, dopaquinone, leukodopachrome, and dopachrome are produced with melanin which is exterminated by the addition of L-ascorbate in similar concentration to tyrosine [87]. Ates [88] used microbial tyrosinase in Cu-alginate gels in continuous and batch systems. The total market volume of L-DOPA is $101 billion per year worldwide (US patent number 5837504, 1998) and thus alternative production methods are still being searched for the production of this drug. The world market for L-DOPA is increasing as indicated by an upliftment of 250 tons per year [89]. In the last few decades, enzymatic production of L-DOPA using microbes from* Erwinia herbicola* has been industrialized [88]. The other microbial sources have been reported earlier for the construction of tyrosinases which convert L-DOPA using as substrate L-tyrosine, including* Erwinia herbicola *[81],* Aspergillus oryzae *[90],* Yarrowia lipolytica *[91],* Acremonium rutilum *[92], and* Bacillus *sp. [93]. Industrial production of L-DOPA by the action of immobilized tyrosinase is shown in Figure 6.

## 10. Industrial Application of Tyrosinase

Chen et al. [94] reported the novel use of tyrosinase for the* in vitro* conjugation of the protein gelatin to the polysaccharide chitosan. Hydroxytyrosol, a potent antioxidant abundant in olives, was also synthesized with tyrosinase from tyrosol. Tyrosinase is used as a potential prodrug for treating melanoma where patients were successfully treated via tyrosinase activity [95, 96]. Anghileri et al. [97] utilize microbial tyrosinase to produce conjugates from sericin, a peptide found in the wastewater of silk textile industries. Recently, EMPA, a transdisciplinary research and service institution, has introduced methods for the recombinant production of bacterial tyrosinase; these are utilized for the formation of biomaterials such as cross-linked proteins and melanin, for example, recombinant* Verrucomicrobium. Spinosum *tyrosinase protein is used to produce tailor-made melanin and other polyphenolic materials using various phenols and catechols as starting materials and these have a wide application as they can be used to develop organic semiconductors or in photovoltaics. The tyrosinase enzyme can also be used to produce cross-linked proteins, allowing enzyme biocatalysts such as lipase to be easily recycled.

## 11. Discovery and Quantification of Phenolic Compounds

In view of the major concerns regarding toxicity, considerable attention has been given to the reliable quantification of phenols in complex environmental matrices. Phenolic compounds are present in the wastewater of a number of industries such as coal conversion, resin and plastics, petroleum refineries, textiles, dyes, iron and steel, and pulp and paper [98]. Phenols are toxic pollutants in industrial wastes imposing several risks on human health and some are suspected carcinogens. In addition, phenol causes coloration of the receiving waters and it is, therefore, essential to decontaminate such compound [99]. Several researchers have studied the use of enzymes in wastewater treatment. The application of a polyphenol oxidase enzyme such as tyrosinase in removal of phenol and its derivatives has become very important and effective method [100]. The tyrosinase from* S. antibioticus*, for example, had activity on industrial pollutants such as 3- and 4-chlorophenols and 3- and 4-fluorophenols [101]. The application of bacterial tyrosinase to the treatment of contaminated wastewaters has recently been reviewed and can be done either with tyrosinase producing stains or with the enzyme in an immobilized form as protagonist [102, 103]. Tyrosinase offers the advantage over other enzyme systems that have been used for phenol removal in that molecular oxygen rather than hydrogen peroxide which is the oxidant, theoretically reducing the potential cost of applying the technology [104]. Among many analytical methods used for fast monitoring of these phenolic compounds, electrochemical biosensors based on immobilized tyrosinase have received the major allocate of attention [105, 106]. Traditionally, spectrophotometric or chromatographic methods are used for the detection of phenolic compounds. Certain new procedures (capillary electrophoresis, immunoassays, and biosensors) have been developed, which potentially provide better specificity, lower costs, and faster and simpler sample processing [107].

Tyrosinases catalyse conversion of phenolic substrate to quinine species that can be electrochemically reduced to allow low potential detection of phenolic analyte [108]. Different methods are utilized for the immobilization of tyrosinase with an electrochemical transducer such as adsorption [109], cross-linking [110], on the surface of electrodes, entrapment in polymer films and hydrogels [111], carbon paste matrix [112, 113] and graphite-epoxy composite electrodes [114]. Electropolymerization [115, 116], self-assembled monolayers [117, 118], silica sol-gel [9], alumina sol-gel [119], and nanoparticles [120] have also been applied for immobilization of tyrosinase for the detection of phenolic compounds. Tembe et al. [121] introduced an electrochemical biosensor for the determination of catechol where the enzyme was entrapped in agarose-guar gum composite biopolymer matrix. The use of the tyrosinase-polysaccharide bioelectrode for monitoring environmental pollution can be initiated as composite material (agarose and guar gum) has a good film forming and adhesion ability, as well as being nontoxic and biocompatible, thus increasing its use for tyrosinase entrapment and subsequent session fabrication. Seetharam and Saville [122] studied the degradation of phenol by tyrosinase immobilized on chemically modified sodium aluminosilicate (NaA), calcium aluminosilicate (CaA), and silica gel which can be reused repeatedly without any decrease in performance. Biosensors based on tyrosinases were designed for measuring phenols, polyphenols, and pesticides. Tyrosinases are also applied in biosensors and microarrays through immobilization [123]; for example, detection of toxic phenolic compounds [124] with consortium to other enzyme (glucose dehydrogenase) [125]. “Class selective” enzyme electrodes based on tyrosinase are used for semiquantitative field screening as well as detectors for liquid chromatography providing quantitation of the individual substrates [126]. Tyrosinase biosensors are also used for monitoring substrate carbonates, pesticides [127], cyanide, organophosphates, or toxic metals [128], where they measure various toxins due to the perturbation and modulation of the enzyme activity. Thus these act as early warning poison detectors.

## 12. Tyrosinase Biosensor Based on Interdigitated Electrodes for Herbicides Determination

In recent year, many amperometric biosensors based on the inhibition of the activity of tyrosinase enzymes have been used for the determination of triazine and phenylurea herbicides in the environment. Use of herbicides is persistent in spite of the danger it causes to the environment, as it is widely used due to its low environmental persistence but it contributes to the high acute toxicity. They cause a potent hazard to human health as their presence can be detected in surface and ground water. Different techniques are used extensively for the detection and quantitative determination of the toxic levels. Typically, liquid chromatography (LC) or gas chromatography (GC) is used but nowadays electrochemical enzyme sensors are also considered an alternative method to the conventional spectrometric techniques for pollutant determination due to their simplified sample. To enhance the biosensor response and sensitivity Fe_3_O_4_ nanoparticles together with immobilized alkaline phosphates into a sol-gel/chitosan biosensor membrane have been incorporated. These nanoparticles used in electrochemical biosensors have the ability to provide a favorable microenvironment for biomolecules such as proteins to exchange electrons directly with an electrode [129]. Similarly, different enzyme based biosensors involving laccase [130, 131], tyrosinase [132], glucose oxidase [133, 134], horseradish peroxidase [135], and Fe_3_O_4_ nanoparticles have been used for the construction of electrochemical biosensors. Tyrosinase electrode is also employed to monitor phenolic and catecholic compounds; it is one of the effective transducers for detection of phenols or catechols [85, 136, 137].

## 13. Manufacturing of Cross-Linked Biopolymers

**Figure 7:**
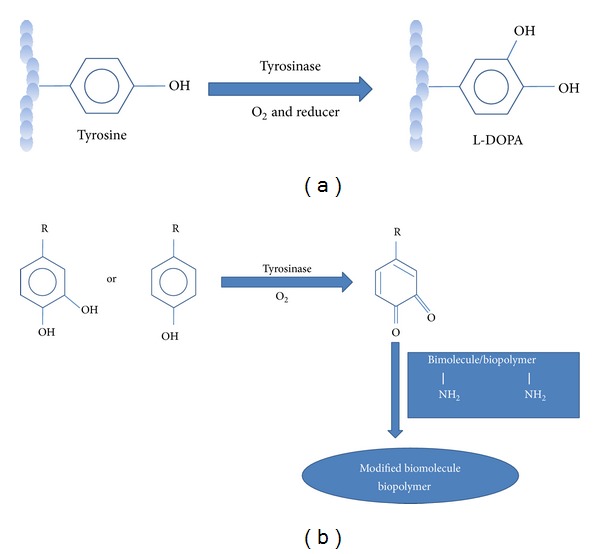
Transfer of tyrosine amino acids into DOPA by the action of tyrosinase in the presence of a reducer. (a) Oxidation of phenol or catechol derivatives into reactive* o*-quinones using tyrosinase, followed by the grafting of amino-functionalized biomolecules/biopolymer (b).

Food industry is continuously developing new biopolymers with special properties not only for their use as emulsifying and thickening agents but also for the production of low-calorie and low-fat foods. Different enzymes are used for hydrolyzing food biopolymers so as to improve product characteristics [138]. Cross-linked biopolymers modify the structural properties of the food matrix [139]. Similarly, enzymatic cross-linking and grafting of specific substances to the biopolymers can be used in the textile industry and for generation of novel biomaterials [129, 140]. The mode of reaction opted by tyrosinase, a cross-linking enzyme for food biopolymers, is direct as it catalyses oxidation of mono- and diphenols to* o*-diquinones utilizing p-coumaric acid (p-CA) and caffeic acid and not ferulic acid (FA) reactive sites in carbohydrates and tyrosine in proteins, respectively [139]. Oxidative enzymes, tyrosinase, have the potential to cross-link food biopolymers [141].* Trichoderma reesei* tyrosinase is found to be an efficient protein cross-linker, when compared to the* T*.* hirsute *laccase or to the tyrosinases of microbial origin. Selinheimo [142] reported the use of tyrosinase and laccase to generate food biopolymers with added functionalities or novel food structures from diverse raw materials. Quinones react nonenzymatically with nucleophilic moieties lysyl, tyrosyl, cysteinyl, and histidinyl residues of proteins [143–145]. They are susceptible to nucleophilic attack by free sulfhydryl and amino groups of amino acid side chains, resulting in formation of tyrosine-cysteine and tyrosine-lysine cross-links in the protein structures [146]. Similarly, they couple to phenolic compounds to form dimeric phenolic conjugates [147]. Desentis-Mendoza et al. [148] reported improved* in vitro* antioxidation by polymerization of phenolic compounds. Oxidation of polyphenols via tyrosinase catalysis has been reported thus improving* in vitro* iron accessibility [149]. The cross-linking of biopolymers using tyrosinase is shown in Figure 7.

## 14. Determination of Certain Compounds in Beverages

Montereali et al. [150] reported the detection of polyphenols present in musts and wines through an amperometric biosensor based on the utilization of tyrosinase and laccase from* Trametes versicolor*. Both enzymes were immobilized on graphite screen-printed electrodes modified with ferrocene. These biosensors exhibited a good sampling behavior compared to that obtained from spectrophotometric analysis but enzymatic activity was prevented due to the presence of SO_2_ [151]. Tyrosinases are also selectively removed byproducts contaminant in industrial fermentation processes [152].

### 14.1. In Cereal Processing

The application of tyrosinases in cereal processing has been well studied as it can catalyze oxidation of phenolic compounds present in cereal proteins and polysaccharides by either producing linkages in or between polysaccharides or in between proteins and polysaccharides or proteins themselves. Kuninori et al. [153] described the effect of tyrosinase (extract of mushroom, rich in polyphenol oxidase) on wheat dough while tyrosinase-catalyzed formation of 2-S-cysteinyl-DOPA, 2,5-di-S-cysteinyl-DOPA, 6-S-cysteinyl-DOPA, 5-S-cysteinyl-3, 4-DOPA, and di-DOPA cross-links has been characterized in gluten proteins [146, 154].

### 14.2. In Dairy Processing

In dairy products, cross-linking can be exploited for prevention of syneresis or to make a soft texture firmer. Hetero-cross-linking or cereal, milk, and meat biopolymers provide a probable means to produce novel food products with precise functionalities and characteristics. Tyrosinase improves functionalization of milk products, for example, tailoring antioxidation of biopolymers. Ito et al. [155] reported oxidation of the tyrosyl residues of dairy proteins whereas Halaouli et al. [20] reported the cross-linking of casein proteins. Furthermore, tyrosinases have been reported to induce partial cross-linking of whey proteins; for example, tyrosinase from* A. bisporus* could cross-link *α*-lactalbumin [156].

### 14.3. In Meat Processing

Cross-linking enzymes play an essential role in tailoring the gelation properties of meat as gel formation ability and textural and binding properties of meat are vital in manufacturing meat products. Tyrosinases have recently been tested for processing of pork and chicken proteins [157, 158]. Tyrosinase effectively improves the gel formation property of a 4% chicken breast myofibrillar protein suspension in presence of 0.35 M NaCl as well as improving the firmness of the homogenate gels containing a lowered amount of meat free of phosphate [159, 160].

## 15. Other Applications

Application of tyrosinase in cell culture has also implicated the wide acceptance of its catalytic property, as it helps nerve cells to grow. The process includes stamping of the tyrosinase enzyme onto plastic surfaces, which causes* in situ* formation of thin films of melanin. It may be useful in the prevention of bacterial contamination, as melanin has a bacteriostatic effect. Immobilization of tyrosinase is used to convert L-tyrosine to L-DOPA by entrapment into either polymer, natural polymer, or modified polystyrene or adsorption on nylon zeolite [161], glass beads, fuller's earth, and chitin activated with hexamethylenediamine [162]. Several studies reported the grafting of silk proteins onto chitosan via tyrosinase reactions specifying tailoring properties of polymers. Likewise, grafting of L-DOPA to wool fibers proteins has also been successfully carried out [163, 164]. It is also used for the preparation of hydrogels for skin substitutes [165], matrices for drug delivery, and tissue engineering [166]. Aberg et al. [129] used tyrosinase for biocatalytic grafting of phenolic moieties or protein onto chitosan. Production of* o*-diphenols was done using mushroom tyrosinase from* Agaricus bisporus *which was immobilized on commercially available epoxy-resin EupergitC250L and then coated using layer-by-layer method (LbL) [167]. The two novel heterogeneous biocatalysts have been characterized for their morphology and reusability. These biocatalysts were used for the efficient and selective synthesis of bioactive catechols under mild and environmentally friendly experimental conditions [168]. Tyrosinase and layer-by-layer supported tyrosinases are also used in the synthesis of lipophilic catechols. A significant antiviral activity has been observed in such derivatives which are characterized by antioxidant activity and long carbon alkyl side chains, suggesting the possibility of a new inhibition mechanism based on both redox and lipophilic properties [169].

## 16. Conclusion

There is an increasing demand for various enzymes in industries, in spite of their wide acceptance in the global market. Tyrosinase enzymes constitute one of the most important groups of commercial enzymes. These enzymes have ample utilization in industrial processes, such as pharmaceuticals and cosmetic and food industries. There are considerable reports indicating the great potential of this enzyme medicine, agricultural industries, and analytical and environmental purposes. It is also used to produce synthetic melanin which provides protection against radiation and is used as cation exchangers, drug carriers, antioxidants, antiviral agents, or immunogens. However, this review shows that microbial tyrosinase is a promising enzyme for pharmaceutical and food bioprocessing technology appraising the state of knowledge about its structure biochemical properties, melanin biosynthetic pathway, purification, and production. We conclude that much more research is necessary in these areas if microbial tyrosinases are to fulfill their industrial potential. In conclusion, more concrete studies of the found microbial tyrosinase with a human clinical point of view are required, and, in our experience, this often needs the help and cooperation of pharmaceutical, cosmetic, or bioprocessing companies.

## Figures and Tables

**Table 1:** Biochemical properties of tyrosinase.

Sources	MW kDa/Da	Optimum temperature	Optimum pH	pI	kM (mM)	Specific activity	References
*Aeromonas media *	58000	50°C	8.0	4.9	0.64	34 µmol/min/mg	[55]
*Beta vulgaris *	41000	25°C	6.0	NR	0.067	NR	[56]
*Lentinula boryana *	20, 27, 47	50°C	6.0	NR	1.9 (L-DOPA)	NR	[14]
*Neurospora crassa *	46	NR	5.0	NR	0.18	540 µmol/min/mg	[9]
*Agaricus bisporus *	112800	25°C	7.0	4.75	0.36 (L-tyrosine)	8300 µmol/min/mg	[57]
*Lentinula edodes *	70, 105	NR	6.5	4.3	0.85 (L-DOPA)	NR	[11]
*Aspergillus oryzae *	67	5.0–6.0	NR	NR	NR	NR	[58]
*Pycnoporus sanguineus *	45000	25°C	6.5	4.55	0.9 (L-DOPA)	3.2 µmol/min/mg	[13]
*Trichoderma reesei *	43.2	30°C	9.0	9.5	7.5 (L-DOPA)	NR	[47]
*Streptomyces glaucescens *	3.09	NR	NR	NR	0.41 (L-tyrosine)		[59]
*Aspergillus nidulans *	50.48	NR	7.0	NR	NR	NR	[60]
*Bacillus megaterium *	31000 35000	50°C	7.0	NR	0.075 (L-tyrosine) 0.35 (L-DOPA)	NR	[41]
*Bacillus thuringiensis *	NR	75°C	9.0	NR	0.563 (L-tyrosine) 0.768 (L-DOPA)	NR	[61]
*Streptomyces *sp.	32000	35°C	7.0	NR	1.25 (L-tyrosine) 4.14 (L-DOPA)	130 µmol/min/mg	[62]
*Pseudomonas putida *	36000 39000	30°C	7.0	NR	0.23 (L-tyrosine) 0.33 (L-DOPA)	0.375 µmol/min/mg	[20]

NR: not reported; DOPA: dihydroxyphenylalanine.
